# International cooperation under the human right to science: What and whose duties and responsibilities?

**DOI:** 10.3389/fsoc.2023.1273984

**Published:** 2023-11-08

**Authors:** Katja Achermann, Samantha Besson

**Affiliations:** ^1^Faculty of Law, University of Fribourg, Fribourg, Switzerland; ^2^Collège de France, Paris, France; ^3^University of Fribourg, Fribourg, Switzerland

**Keywords:** international cooperation, human right to science, duties, responsibilities, institution-building

## 1. Introduction

States Parties to the 1966 International Covenant on Economic, Social and Cultural Rights (ICESCR) have explicitly recognized the “benefits to be derived from the encouragement and development of international contacts and co-operation in the scientific field” in Article 15(4) of the Covenant.

In its General Comment No. 25 published in 2020, the Committee on Economic, Social and Cultural Rights (CESCR) posits that this provision “reinforces” the general “duty to cooperate internationally toward the fulfillment of all economic, social and cultural rights” as established in Article 2 ICESCR and in Articles 55 and 56 of the United Nations (UN) Charter (Committee on Economic, Social and Cultural Rights, 2020, paras. 77–84). Based on this reinforced duty of international cooperation, States have to promote an “enabling global environment for the advancement of science and the enjoyment of the benefits of its applications”, by taking steps through “legislation and policies, including diplomatic and foreign relations” in the scientific realm (Committee on Economic, Social and Cultural Rights, 2020, para. 77).

According to General Comment No. 25, this “reinforced duty of international cooperation”^1^ has four important “justifications” that also amount to four “dimensions” of the duty.^2^

First, to the extent that scientific progress in certain fields requires universal endeavor, States should encourage international cooperation among scientists, including through their participation in the “international scientific and technological community”.^3^ Second, due to deep international disparities in science and technology, complying with their respective duties under the ICESCR requires “developed States” to assist “developing States” and cooperate with them in order to contribute to the “development of science and technology” in these States.^4^ Conversely, and in priority actually, developing States should resort to international assistance and cooperation with developed States. Third, subject to “due incentives and regulations”, benefits and applications of scientific progress should be “shared with the international community”, including “developing countries”, “communities living in poverty” and “groups with special needs and vulnerabilities”, especially when those benefits are related to the enjoyment of economic, social and cultural rights.^5^ Finally, to the extent that the harms and risks of harm related to science and technology in many areas are “transnational”, efforts to prevent such harms or mitigate their effects require the international cooperation of States, including through “multilateral agreements”.^6^

One should commend the CESCR for the detailed treatment of the “duty to cooperate internationally” in General Comment No. 25. It is not only a timely tribute to the importance of the duty of international cooperation for the “full realization of the right to participate in and to enjoy the benefits of scientific progress and its applications”^7^ (RPEBSPA) or, for short, the “human right to science” guaranteed by Article 15(1)(b) ICESCR.^8^ It is also a first much needed attempt at fleshing out the grounds, content and bearers of that duty as one of the many duties arising under the RPEBSPA.^9^ However, one cannot help but also reading it as a missed opportunity. Even though it dedicates two pages out of nineteen to the duty, the CESCR does not succeed in dispelling the indeterminacies that have long plagued the duties and responsibilities^10^ of cooperation, both in international human rights law in general^11^ and relating to the RPEBSPA in particular (Chapman, 2009; Müller, 2010). It arguably even makes some of them worse.

Drawing on its two authors' previous publications on this issue,^12^ this opinion piece assesses the CESCR's understanding of the reinforced duty of international cooperation under the RPEBSPA in General Comment No. 25 and makes different proposals to improve it. In the first section, it identifies three indeterminacies in the CESCR's understanding of the duty. The second section points to two ways in which these indeterminacies could be resolved in future practice.

## 2. Three critiques

There are three indeterminacies surrounding the duty of international cooperation under the RPEBSPA in General Comment No. 25. They pertain to: the legal bindingness of the duty; its content; and its bearers and their relations.

### 2.1. The legal bindingness of the duty of international cooperation

General Comment No. 25 does little to resolve the continuing indeterminacy surrounding the existence of a legal duty and/or responsibility of international cooperation under the RPEBSPA, but also under the ICESCR more generally. The terms used in Articles 2(1) and 15(4) ICESCR are notoriously unclear on this point (Alston and Quinn, 1987, 186–192).

True, the CESCR starts by boldly placing Articles 2(1) and 15(4) ICESCR and Articles 55 and 56 UN Charter on the same plane and by drawing what it refers to as a single “reinforced duty of international cooperation” expressly from their conjunction.^13^ This is a welcome clarification and assertion. However, in its subsequent elaborations on the measures to be adopted to comply with this identified duty of international cooperation under the RPEBSPA, the CESCR consistently resorts to the word “should” and does not identify further specific “duties” and/or “responsibilities” to cooperate internationally.^14^ The terminology only changes in the two final paragraphs of the General Comment's section on international cooperation when the CESCR refers to the “extraterritorial obligations” States *also* incur under the RPEBSPA.^15^ It is difficult to know, however, whether the “also” used there implies that the other “dimensions” of the duty to international cooperation presented earlier are “obligations” or “duties” as well or that the only legal obligations or duties incurred as obligations of international cooperation are the extraterritorial obligations discussed in those two paragraphs.

So-doing, the CESCR misses the opportunity not only to clearly establish, once and for all, the legal bindingness of the duty of international cooperation it grounds in the RPEBSPA, but also to spell out the multiple specific duties of international cooperation that correspond to the four “dimensions” of the duty it distinguishes.

Moreover, the Committee also contributes to the conflation between the transnational or international dimension of the duty of cooperation with other States that arises under the RPEBSPA, on the one hand, with the extraterritorial application of that duty, on the other. Most extraterritorial obligations are not duties of international cooperation, however. They require a State to act individually to protect human rights when it has effective control over right-holders situated on another State's territory.^16^ As a matter of fact, States' duties to cooperate internationally are not necessarily extraterritorial either. Indeed, many duties to cooperate with others may be considered to arise under any given State's territorial jurisdiction, and not only under its extraterritorial jurisdiction.^17^

### 2.2. The content of the duty of international cooperation

A second feature in the CESCR's treatment of the duty of international cooperation in General Comment No. 25 is its lack of specificity on the content of that duty.

True, the Committee is quite concrete in relation to the first and the fourth “justifications and dimensions” of international cooperation in the scientific realm. In relation to nurturing science as a universal endeavor, the CESCR comments that States should promote and enable scientists' participation in the international scientific and technological community, *inter alia* by facilitating air travel, data sharing arrangements and accessibility of educational resources (see text footnote 2). In order to prevent and mitigate the potentially harmful effects of science and technology and their applications on a transnational scale, the Committee posits that States “should promote multilateral agreements” to organize robust international cooperation.^18^ However, the CESCR fails to specify the content of the duty of international cooperation in each of those two cases.

Moreover, with respect to the third dimension of the duty of international cooperation that relates to the sharing of the benefits and applications of scientific progress with the international community, the CESCR refrains from specifying the content of the relevant duty.^19^ As to the second dimension of the duty of international cooperation relating to the scientific disparities between States, it is reduced tautologically to “resort[ing] to international assistance and cooperation” (see text footnote 4).

More generally, one may quibble about the choice of the term “dimensions” of the duty of international cooperation. It may have been better to refer to four actual “types” of duties of international cooperation. The fact that the Committee also refers to those four dimensions as “justifications” is indeed a clear indication of the need to ground them normatively as duties under the RPEBSPA and hence to link their content to their object of protection. In any case, those four dimensions of the duty of international cooperation overlap (e.g., accessing to and sharing scientific benefits under the second and third dimensions of the duty), and it is unclear whether they are exhaustive of international cooperation in the field of science. It remains to be determined, moreover, whether all duties arising under the RPEBSPA also have a cooperative dimension, including an international one.

Last but not least, one particularly striking omission of the General Comment in this regard is its failure to engage with the positive cooperative duty to build international institutions for scientific cooperation with other States. That duty was mentioned as early on as 2009 by the UNESCO's Venice Statement as the duty “to establish institutions to promote the development and diffusion of science and technology”^20^ and was actually considered to be the overarching duty to fulfill corresponding to the RPEBSPA.^21^

### 2.3. The bearers of the duty of international cooperation

A third indeterminacy relates to the question of who should cooperate with whom, that is of the bearers of the duty of international cooperation under the RPEBSPA.

In relation to the second justification for the duty of international cooperation, i.e. the disparities among States in science and technology, the CESCR distinguishes between “developing” and “developed” States.^22^ Beyond this rather crude distinction, however, the Committee fails to systematically reflect on the identity of the public institutions bearing the duty. One may wonder, for instance, whether the duty-bearers do not only include States, but also international organizations (IOs)—the Committee only stresses, in this respect, that IO Member States still bear their human rights duties.^23^ Furthermore, despite noting that “a significant proportion of scientific research is carried out by business enterprises and non-state actors”,^24^ the Committee does not explain how private persons and institutions could be bound, directly or indirectly, by the duty of international cooperation—again, the Committee merely emphasizes States' due diligence duties to regulate and monitor them when they have control over them.^25^

Importantly, by identifying a single “duty” of international cooperation owed under Article 2(1) ICESCR and Articles 55 and 56 UN Charter and one that is “reinforced” by Article 15(4) ICESCR, the CESCR fails to distinguish between the “duties” of international cooperation *stricto sensu* and the “responsibilities” of international cooperation.^26^ Whereas human rights duties, including duties of cooperation, are owed to the right-holders of the RPEBSPA and are grounded in the duty-bearing States' (territorial or extraterritorial) jurisdiction *qua* effective control over them, responsibilities for human rights are not owed to the right-holders, but to other States as *erga omnes partes* duties and arise under all ICESCR rights including the RPEBSPA. More precisely, the latter include responsibilities to cooperate and assist the duty-bearing States in complying with their own (jurisdictional, territorial and extraterritorial) duties under the RPEBSPA.

Failing to distinguish between those duties and responsibilities to cooperate internationally prevents the Committee from addressing the multiplicity of duty- and responsibility-bearers of international cooperation under the RPEBSPA. More specifically, it cannot distinguish between the duty-bearing public institutions, such as States and IOs, and the additional and concurrent responsibility-bearers. The latter include States or IOs that do not have (territorial or extraterritorial) jurisdiction over the respective right-holders, but also private persons (e.g., scientists and researchers) or institutions (e.g., private research foundations or multinational corporations).^27^

By extension, General Comment No. 25 is also silent on the allocation of duties and responsibilities between those multiple cooperation duty-bearing States and/or public institutions, on the one hand, and between them and other responsibility-bearers, on the other. The CESCR merely begs the question when it refers to securing persons in other States an “adequate” access to scientific results (see text footnote 22). Here again, one may regret the lack of concern by the CESCR for the institutionalization of international cooperation and for how international institutions could help co-specify and co-allocate duties and responsibilities among the multiple duty-bearers and/or responsibility-bearers.^28^

## 3. Two proposals

In response to those three critiques of the General Comment No. 25, this section makes two proposals to dispel the indeterminacies surrounding the duty of international cooperation arising under the RPEBSPA: grounding the separate legal duties and responsibilities of international cooperation; and building international institutions to co-specify and co-allocate duties and responsibilities between their multiple bearers.

### 3.1. Grounding the legal duties and responsibilities of international cooperation

To dispel the first indeterminacy criticized in Section 2.1, it is important to ground, i.e., justify, the separate legal duties and responsibilities of international cooperation.

One way of doing so is to ground them specifically in the international public good of science. As one of this piece's authors has argued elsewhere, indeed, a key specificity of the duties and responsibilities corresponding to the RPEBSPA stems from their pertaining to a public, participatory and communal good. Their bearers do not merely owe them individually as they would were the protected interest individual only, but also bear those duties collectively. What this means is that States and IOs owe their respective individual duties and responsibilities relative to the RPEBSPA *together*.^29^ The resulting collective dimension of all duties and responsibilities arising under the RPEBSPA also implies a cooperative one.

More specifically, the justification of the collective and hence cooperative dimension of the *duties* arising under the RPEBSPA is two-pronged: it resides in the universal scope of the public good of science and, by extension, in the universal scope of the standard threats to the interests in that good.^30^ As a right to a public good that is universal,^31^ first, the human right to participate in science can only be effectively protected if all its duty-bearers worldwide, i.e. primarily States and IOs, cooperate in specifying, allocating and fulfilling together the duties they bear separately and individually toward the right-holders under their respective (territorial and extraterritorial) jurisdiction.^32^ Second, the cooperative nature of those duties is also a condition of the feasibility of the protection of their right-holders' interests against threats to the public good of science since those threats have become global or, at least, transnational. Cooperation in the co-specification, co-allocation and co-fulfillment of the duties arising under the RPEBSPA conditions moreover the overall fairness of the burden of those duties on each of the duty-bearing State or IO with (territorial or extraterritorial) jurisdiction.

The proposed justification of the legal duties of international cooperation arising under the RPEBSPA is consistent with the General Comment No. 25′s reference to science as “universal endeavor”. It also fits and justifies the distinction the CESCR makes between different “dimensions” of the duty of international cooperation. It seems, indeed, that three of the four dimensions of the duty of international cooperation identified by the CESCR correspond to the three dimensions or duties one usually identifies as arising under the RPEBSPA:^33^ the right to participate in the scientific enterprise and its organization (matched by the first dimension of the duty of international cooperation, i.e., strengthening participation in the international scientific and technological community worldwide), but also the right to access to and to enjoy the benefits of scientific progress (corresponding to the third dimension of the duty of international cooperation, i.e., sharing the benefits and applications of scientific progress among members of the international community) and the right to be protected against the adverse effects of science (matched by the fourth dimension of the duty of international cooperation, i.e., preventing the transnational harms of scientific progress and mitigating their effects together).

The same may be argued for the *responsibilities* for the RPEBSPA that should be regarded as cooperative in the same way as the corresponding duties. It is the pivotal importance of those responsibilities to cooperate for the human right to participate in the international public good of science that explains the separate and explicit, and hence “reinforced” reference to “international assistance and cooperation” in Article 15(4) ICESCR.^34^ Strictly speaking, however, what is at stake here are “supporting” (UN Human Rights Council, 2012) responsibilities for the right to participate in science bearing on all States parties to the ICESCR at once, and not only on any given State of (territorial or extraterritorial) jurisdiction.^35^

The proposed interpretation of the responsibilities to cooperate grounded in the RPEBSPA is consistent with the second “dimension” of the “duty” of international cooperation justified by the General Comment No. 25 relating to existing “international disparities in science and technology”. Although the CESCR does not draw a distinction between duties and responsibilities of international cooperation in that passage, it tautologically identifies the second dimension of the duty of international cooperation with the “resort to international cooperation and assistance”, thereby hinting at its generality. One may therefore consider that what the CESCR refers to there is actually a “responsibility” of States to assist each other and to cooperate in order to contribute to the “development of science and technology” in all States.

### 3.2. Building international institutions to co-specify and co-allocate duties and responsibilities of cooperation

In order to address the second and third indeterminacies identified in Sections 2.2. and 2.3., our second proposal, when specifying the content of the duties and responsibilities of international cooperation, is to focus on the general positive duty of institution-building mentioned earlier, i.e., the positive duty to institutionalize what the CESCR refers to as the “international scientific and technological community”.^36^

While all the manifestations of international cooperation mentioned by the CESCR correspond to what this piece has argued could amount to the content of the duties under or responsibilities for the RPEBSPA, they barely scratch the surface of what is required in terms of international cooperation in order to effectively realize the right in all its facets. Respect for those duties and responsibilities cannot be exhausted in the adoption of “multilateral agreements”, for instance. Rather, and most fundamentally, it includes duties and responsibilities to engage in international institution-building (Maastricht Principles on Extraterritorial Obligations of States in the Area of Economic, 2011, paras. 77–84). The organization of such an international institutional framework for science may also be what the CESCR means when it refers to promoting an “enabling global environment for the advancement of science and the enjoyment of the benefits of its applications” (see text footnote 13). This includes furthering “diplomatic and foreign relations” better known under the name of “science (and) diplomacy”, but it also implies taking international scientific cooperation to the next, more institutional level.^37^ That international institutional framework, however, still remains to be developed.

As one of this piece's authors has argued elsewhere, the justification for such a positive duty and responsibility of international institution-building under the RPEBSPA lies in the participatory and communal dimensions of the public international good of science.^38^ Indeed, the “participatory” practice of science requires cooperation and organization, and hence some form of public institutionalization and legalization. This is even more the case of participatory goods that are also “communal” ones, like science, i.e., that trigger a common responsibility on the part of those who participate in the practice. Such a common responsibility requires public institutional channeling and legal mediation to specify and allocate individual and collective responsibilities. To the extent that good science needs to be universal, as confirmed by the CESCR, such a universal participatory and communal practice cannot be organized unless it is also institutionalized internationally, i.e., outside of the legal orders of individual States, but also outside of the order of the global market.

In turn, and as mentioned earlier, those international institutions could also help co-specify and co-allocate the duties and responsibilities under the RPEBSPA among their multiple duty-bearers and/or responsibility-bearers (see text footnote 28). International institutions are required in particular in order to distribute the burden of international cooperation in the scientific realm more fairly and effectively. This extends, for instance, to the determination of the “adequate” level of access to scientific results across the “international community” which the CESCR rightly advocates, but does not further explicate. Such institutions could also contribute, more generally, to the co-specification of another pivotal albeit underdetermined principle applicable to participation in the universal practice of science, i.e., equality.^39^

## Author contributions

KA: Writing—original draft. SB: Writing—original draft.

## Footnotes

^1^For an earlier reference to the “need to strengthen international cooperation”, see UN Human Rights Council Special Rapporteur Farida Shaheed (2012), para. 67.

^2^Committee on Economic, Social and Cultural Rights (2020), para. 78.

^3^*Ibid*.

^4^*Ibid*, para. 79.

^5^*Ibid*, para. 80.

^6^*Ibid*, para. 81.

^7^*Ibid*, para. 11.

^8^On the latter now common but problematic short denomination of the right, see e.g., Besson (2015c, 2023b).

^9^Committee on Economic, Social and Cultural Rights (2020), para. 52. For earlier discussions of international cooperation, see UN General Assembly (1975), paras. 1 and 5; UNESCO (2009), paras. 66-68 and 70-73.

^10^On this distinction, see Besson (2015b). See also section 2.2. below.

^11^For an interpretation of the possible grounds, content and bearers of the duties of cooperation under international human rights law, see e.g. Mayer (2021); further Achermann (2022), on file with the authors.

^12^On duties of cooperation under international human rights law: Achermann (2022) (1); on duties and responsibilities of cooperation under the RPEBSPA (Besson, 2015a).

^13^Committee on Economic, Social and Cultural Rights (2020), para. 77.

^14^*Ibid*, paras. 78–82.

^15^*Ibid*, paras. 83 and 84.

^16^On extraterritorial human rights obligations in general, see Besson (2012) and Besson (2023a, 270–292). On the further distinction between a State's extraterritorial jurisdiction *qua* effective control over right-holders situated on another State's territory and a State's reasonable control over a potential source of harm *qua* condition for the due diligence standard to apply, see Besson (2020).

^17^See Besson (2015a), 472-3, 484; Besson (2023b).

^18^*Ibid*, paras. 81 and 74, see also Besson (2023c).

^19^Committee on Economic, Social and Cultural Rights (2020), para. 80.

^20^See UNESCO (2009), paras. 4, 16(a) and 16(d). For earlier discussions, see UN General Assembly (1975), paras. 1 and 5; or UN Human Rights Council Special Rapporteur Farida Shaheed (2012), paras. 67 and 75. Contrast with Committee on Economic, Social and Cultural Rights (2020), para. 52.

^21^See Müller (2010), 779-83; Chapman (2009), 24-27 and 29-31; Besson (2015a), 481-482; Besson (2023b).

^22^Committee on Economic, Social and Cultural Rights (2020), para. 79.

^23^*Ibid*, para. 83.

^24^*Ibid*, para. 58.

^25^*Ibid*, para. 84.

^26^On this distinction in general, see Besson (2015b). On the distinction in the context of the RPEBSPA, see Besson (2015a), 469–470.

^27^See Besson (2015a), 470–476.

^28^For a full argument, see Besson (2015a), 479-482; Besson (2023b).

^29^See Besson (2023b); further Besson (2015b).

^30^See Besson (2015a), 477-478.

^31^See UNESCO (2017), paras. 1 and 18–23.

^32^See UNESCO (2009), para. 4.

^33^See UN Human Rights Council Special Rapporteur Farida Shaheed (2012), para. 25 et seq.; further UNESCO (2009), paras. 13(a), (b) and (c).

^34^See e.g., UN Human Rights Council Special Rapporteur Farida Shaheed (2012), para. 68.

^35^See also Müller (2010), 781-82; Besson (2015a), 470 and 477-478; Besson (2015b), 262.

^36^See Committee on Economic, Social and Cultural Rights (2020), para. 78, by reference UNESCO (2017), para. 31.

^37^See Besson (2015a), 481–482.

^38^Besson (2023b).

^39^Committee on Economic, Social and Cultural Rights (2020) focuses on “discrimination” in the domestic context only, see paras. 17, 25–27 and 28–38, and then only refers to the “deep international disparities among countries in science and technology” in a single paragraph, namely para. 79.
